# Off-label uses of Aflibercept, Ranibizumab and Dexamethasone implant for diabetic retinopathy in Turkey

**DOI:** 10.22336/rjo.2022.56

**Published:** 2022

**Authors:** Mevlut Yilmaz, Mehmet Citirik, Hanife Rahmanlar, Ali Alkan, Hakki Gursoz

**Affiliations:** * Department of Ophthalmology, University of Health Sciences, Ankara Ulucanlar Eye Training and Research Hospital, Ankara, Turkey; ** Turkish Medicines and Medical Devices Agency, Turkish Ministry of Health, Ankara, Turkey

**Keywords:** aflibercept, dexamethasone implant, diabetic macular edema, diabetic retinopathy, off-label drug, ranibizumab

## Abstract

**Objective:** Diabetic retinopathy (DRP) is the most common retinal vascular disease leading to blindness. There is limited data about the off-label drug use for DRP and diabetic macular edema (DME) in literature. The aim of the article was to evaluate the applications for off-label drug use in patients with DME and DRP in Turkey in terms of demographic and clinical characteristics.

**Methods:** Applications for off-label drug use from hospitals across Turkey to the Turkish Medicines and Medical Devices Agency for DRP in 2018 were reviewed retrospectively.

**Results:** 112 approved applications for 167 eyes were included in our study. The mean age of the cases was 61.24 ± 10.23 years, of them 57.1% were males and 42.9% were females. Of these applications, 41.1% were for aflibercept (n:46), 33.9% for ranibizumab (n:38), and 25% for dexamethasone implant (n:28). There was no application for bevacizumab. In terms of referring hospitals, public university hospitals were in the first place with a rate of 70.5%. The most common reasons for applications were drug switchback request and failure to complete loading dose, respectively.

**Discussions:** DRP treatment can sometimes be challenging. The effectiveness of the intravitreal drugs may decrease over time and drug switching may be necessary. In Turkey, intravitreal drugs are only approved and reimbursed for DRP patients in case of macular edema. Off-label drug use may be preferred in non-approved indications and for reasons such as the need for additional drug doses to the determined limits. However, permission must be obtained from TMMDA for off-label drug use in Turkey.

**Conclusion:** Anti-vascular endothelial growth factor drugs are the first-line treatment options for DME. TMMDA currently approves stepwise therapy for diabetic macular edema, initiated with bevacizumab. Bevacizumab administration does not require approval for off-label application. Additionally, ranibizumab, aflibercept, and dexamethasone implant are reimbursed only in case of failure to respond to 3 doses of bevacizumab injection. Our report provides information about off-label drug preferences and drug use regulations in DRP treatment in Turkey.

**Abbreviations:**DME = diabetic macular edema, DRP = Diabetic retinopathy, FFA = fundus fluorescein angiography, TMMDA = Turkish Medicines and Medical Devices Agency, VEGF = vascular endothelial growth factor

## Introduction

Diabetic retinopathy (DRP) is a common cause of blindness worldwide and imposes a severe burden on healthcare systems [**1**,**2**]. In healthy people, the passage of fluid and other molecules between the blood and retina, the layer of the eye that contains light-sensitive photoreceptor cells and nerve cells, is kept under control by means of special barrier layers [**3**]. Scientific evidence indicates that in DRP patients, deterioration in blood flow due to hyperglycemia-related retinal vascular changes, and resultant ischemia in the retina trigger the release of vascular endothelial growth factor (VEGF) and various inflammatory mediators [**1**,**4**]. These mediators induce increased vascular permeability in the retina, retinal/ disc neovascularization, intraretinal/ intraocular hemorrhages, and ultimately vision loss [**4**,**5**].

Anti-VEGF monoclonal antibody drugs (bevacizumab, ranibizumab, and aflibercept) are the first-line therapy choices in the treatment of diabetic macular edema (DME). In addition, intravitreal steroid implants can be applied when clinically necessary due to their anti-inflammatory/ antiangiogenic effects [**5**]. Ranibizumab (Lucentis, Genentech Inc., San Francisco, USA) was the first agent licensed for the treatment of DME. It consists of the antigen-binding part of the anti-VEGF antibody (the Fab fragment), which binds to all isoforms of VEGF-A [**6**]. Aflibercept (Eylea, Bayer AG, Berlin, Germany) is a recombinant fusion protein created by combining the extracellular portions of VEGF 1 and 2 receptors to the Fc fragment of the Anti-VEGF antibody. It blocks VEGF-A, VEGF-B, and placental growth factor [**6**,**7**]. Dexamethasone implant (Ozurdex, Allergan Inc, California, USA) is a potent corticosteroid that has been proven to reduce inflammation and central macular thickness, and improve vision in DRP patients [**8**]. These drugs are approved for DME in Turkey.

Until the end of 2018, the principles of drug use and reimbursement rules in retinal vascular diseases and diabetic macular edema in Turkey were different from the current rules in some respects. Physicians could start initial treatment with ranibizumab, aflibercept, or dexamethasone implant. For anti-VEGF drugs, a loading dose of at least three doses with an interval of four to six weeks was mandatory. If three-dose loading therapy was not completed and interrupted longer than required, it was necessary to complete the loading dose as three doses, again starting from the beginning. Otherwise, an application for off-label drug use was required. Only one switch was allowed between anti-VEGFs. For re-switches (switchback) between medications, off-label use application was required. Dexamethasone implant use was reimbursed for a maximum of two doses per year and up to seven doses in total. Additional board approval was required for uses beyond these limits. Licensed anti-VEGF drugs were indicated only for DRP cases with macular edema.

Since the beginning of 2019, the practice of drug use in DME has changed and the transition to other licensed drugs has been allowed after 3 doses of off-label intravitreal bevacizumab injections. The switchback restriction was lifted, however, for switching from ranibizumab to aflibercept or vice versa, three doses of bevacizumab loading dose were stipulated in between. The annual application limit for the dexamethasone implant has been increased to four doses and the total dose restrictions have been lifted.

Authorized drug regulatory institutions determine criteria for the drugs they license, such as dose, frequency, age group, route of administration, and indications. Drug use that does not meet these criteria refers to off-label drug use [**9**,**10**]. As far as we know, there is no data in the English literature regarding the off-label use of approved drugs such as ranibizumab, aflibercept, and dexamethasone implant in DRP. In this study, we aimed to investigate the clinical and demographic characteristics of off-label drug applications made to the Turkish Medicines and Medical Devices Agency (TMMDA) for DME and DRP. Bevacizumab was not evaluated in this study since it is included in the list of off-label drugs used without permission in our country.

## Materials and methods

In this clinical study, the applications for the off-label use of anti-VEGF drugs (aflibercept, ranibizumab, and dexamethasone implant) from hospitals across Turkey to TMMDA in 2018 were retrospectively evaluated.

Written permission from TMMDA and approval from the Ethics Committee of Dışkapı Training and Research Hospital were obtained before the study. This study was conducted in accordance with the principles of the Declaration of Helsinki. The application files were reviewed in terms of demographic and clinical data such as previous treatment regimens, requested drugs, reasons for applications, type of applicant hospitals, and the results of applications.

Data were analyzed using the IBM Statistical Package for Social Sciences version 22 (IBM SPSS Statistics for Windows, Version 22.0. Armonk, NY: IBM Corp). The distribution of data was evaluated using the Kolmogorov-Smirnov test. Descriptive statistical analyzes (frequency, means, standard deviations, etc.) were recorded. The Chi-square test or Fisher’s exact test was used to compare categorical variables.

## Results

Our study included approved off-label drug use applications for 167 eyes of 112 patients. Of all the cases, 64 were males (57.1%) and 48 were females (42.9%). The mean age of the patients was 61.24 ± 10.23 (**Table 1**). The diagnosis of all patients was DRP/ DME. Applications were most frequently made for aflibercept (n:46, 41.1%), ranibizumab (n:38, 33.9%), and dexamethasone implant (n:28, 25%) (**Table 1**). There were no significant differences between these drugs. However, when the sum of ranibizumab and aflibercept was evaluated as a group (n:84), anti-VEGF drug requests were found to be significantly higher than dexamethasone requests (n:28) (p<0.001, chi-square test).

**Table 1:** Demographic characteristics of the applications

	Ranibizumab	Aflibercept	Dexamethasone implant	Total
Number and Percentage	38 (33,9%)	46 (41,1%)	28 (25%)	112
Age (mean ± std dev.)	57,53 ± 10,74	61,41 ± 9,18	66 ± 9,46	61,24 ± 10,23
Sex (M/ F)	20/ 18	30/ 16	14/ 14	64/ 48
Laterality (Right/ Left/ Bilateral)	11/ 11/ 16	16/ 5/ 25	6/ 8/ 14	33/ 24/ 55

The reasons for applications were as follows: for aflibercept (n:46), switchback (ranibizumab use after aflibercept and switching to aflibercept again) (n:24, 52.2%), failure to complete the loading dose due to patient-related reasons (n:9, 19.6%), persistent neovascularizations despite laser photocoagulation (n:5, 10.9%), failure to take fundus fluorescein angiography (FFA) (n:4, 8.7%), and pre-vitrectomy (n:4, 8.7%) (**Table 2**).

The reasons for the applications for ranibizumab (n:38) were as follows: switchback (aflibercept use after ranibizumab and switching to ranibizumab again) (n:23, 60.5%), persistent neovascularizations despite laser photocoagulation (n:6, 15.8%), failure to complete the loading dose due to patient-related reasons (n:6, 15.8%), pre-vitrectomy (n:2, 5.3%), and failure to take FFA (n:1, 2.6%) (**Table 2**). 

There were no significant statistical differences between ranibizumab and aflibercept in terms of reasons for applications (p:0>0,05 for all, Fisher’s exact test).

The reasons for application for dexamethasone implant (n:28) were as follows: 7 dose limit (n:15, 53.6%), 2 drugs limit per year (n:11, 39.3%) and failure to take FFA (n:2, 7.1%) (**Table 2**).

Public university hospitals (n:79, 70.5%) (44.3% Aflibercept, 34.2% Ranibizumab, 21.5% Dexamethasone implant) ranked first in the applicant hospitals (p<0,001), followed by training and research hospitals (n:24, 21.4%) (41.7% Ranibizumab, 33.3% Aflibercept, 25% Dexamethasone implant), and foundation university hospitals (n:9, 8.1%) (55.5% Dexamethasone implant, 33.3% Aflibercept, 11.1% Ranibizumab) respectively. There was no difference in the type of requested drugs according to hospital types (p>0,05).

**Table 2:** Reasons for the applications

	Ranibizumab (Number, %)	Aflibercept (Number, %)	Dexamethasone implant (Number, %)	Total (Number, %)	General Total (Number, %)
Switchback (Number, %)	23	26	0	49	49
	46,9%	53,1%	0,0%	100,0%	43.7%
Non-completion of loading dose due to patient-related reasons, (Number, %)	6	9	0	15	15
	40,0%	60,0%	0,0%	100,0%	13.4%
7 dose limit (Number, %)	0	0	15	15	15
	0,0%	0,0%	100,0%	100,0%	13.4%
NV1 despite LPC2 (Number, %)	6	5	0	11	11
	54,5%	45,5%	0,0%	100,0%	9,8%
More than 2 drug requirements per year (Number, %)	0	0	11	11	11
	0,0%	0,0%	100,0%	100,0%	9,8%
Pre-vitrectomy (Number, %)	2	4	0	6	6
	33,3%	66,7%	0,0%	100,0%	5,4%
Failure to do FFA3 (Number, %)	1	2	2	5	5
	20,0%	40,0%	40,0%	100,0%	4,5%
Total (Number, %)	38	46	28	112	112
	33,9%	41,1%	25,0%	100,0%	100,0%
1NV = neovascularization 2LPC = laser photocoagulation 3FFA = fundus fluorescein angiography					

The treatment regimens before applications are presented in **Table 3**. Accordingly, drug applications were made most frequently after the use of both ranibizumab and aflibercept (33%), and after the use of all three - ranibizumab, aflibercept, and dexamethasone implant (21.4%).

**Table 3:** Treatment regimens before application

	Ranibizumab (Number, %)	Aflibercept (Number, %)	Dexamethasone implant (Number, %)	Total (Number, %)	General Total (Number, %)
Ranibizumab + Aflibercept	16	19	2	37	37
	43,2%	51,4%	5,4%	100,0%	33%
Dexamethasone implant + Ranibizumab + Aflibercept	8	11	5	24	24
	33,3%	45,8%	20,8%	100,0%	21.4%
Dexamethasone implant + Ranibizumab	3	4	9	16	16
	18,8%	25,0%	56,3%	100,0%	14,3%
Dexamethasone implant	2	1	12	15	15
	13,3%	6,7%	80,0%	100,0%	13,4%
Ranibizumab	4	7	0	11	11
	36,4%	63,6%	0,0%	100,0%	9,8%
Aflibercept	5	3	0	8	8
	62,5%	37,5%	0,0%	100,0%	7,1%
Dexamethasone implant + Aflibercept	0	1	0	1	1
	0,0%	100,0%	0,0%	100,0%	0,89%
Total	38	46	28	112	112
	33,9%	41,1%	25,0%	100,0%	100%

## Discussions

According to the results of our study, the most requested off-label drugs for DRP were anti-VEGFs. The most common reasons for applications were the switchback for anti-VEGF drugs and dose limitations for dexamethasone. The reasons for application were similar for both anti-VEGFs. Drugs are reimbursed only for macular edema in DRP patients in Turkey. Off-label utilization reasons other than DME were: pre-vitreoretinal surgery (vitrectomy) to reduce the risk of bleeding, persistent neovascularizations despite laser photocoagulation, failure to complete the loading doses due to patient-related reasons, and failure to take fundus fluorescein angiography (FFA) before the injection. The reasons for not completing the loading dose were the patients’ non-compliance with the medical follow-up recommendations, often due to comorbidities and socioeconomic reasons.

For off-label drug use, there must be some reasonable justifications, such as inadequate response to the licensed drug, socioeconomic status of the patients, and the fact that newly released alternative drugs have not yet been licensed [**11**]. In such cases, an official application can be made online, to TMMDA, for off-label drug use in Turkey. If the use of the requested drug is deemed appropriate by the scientific review board, the drug is temporarily included in the scope of reimbursement for that patient and can be prescribed. However, for some drugs, such as bevacizumab, off-label use is possible without obtaining permission from TMMDA [**12**,**13**]. According to a report from the USA, bevacizumab costs approximately 30 times less per dose than aflibercept and 20 times less than ranibizumab in the treatment of DME [**14**]. Bevacizumab is frequently used off-label in ophthalmology due to its cost-effectiveness, easy accessibility, and no need to apply for off-label use [**15**]. Since there is no need to obtain permission for its use in Turkey, it is difficult to reach clinical data on its use in ophthalmology. Therefore, in this study, information regarding the use of intravitreal bevacizumab in diabetic retinopathy could not be presented.

In addition to increased VEGF levels, enhanced inflammation is also a key factor in the pathogenesis of DRP. Various inflammatory cytokines such as TNF-α, Prostaglandin E2, IL-1β, and chemokines increase in the retina and vitreous, and microglial cell activation enhances neuroinflammation [**16**]. Recent studies report that especially the presence of intraretinal hyperreflective dots and serous macular detachment is associated with inflammation and intravitreal corticosteroid injections are beneficial in improving these findings [**17**]. However, intravitreal steroid injections are not generally preferred as first-line therapy because they accelerate the development of glaucoma and cataracts [**18**]. Dexamethasone implant is the only corticosteroid licensed for DME treatment in Turkey. With a single intravitreal injection, the implant releases rapidly for the first 2 months and slowly for up to 3 months and can provide treatment for an average of 3-4 months [**8**]. It strengthens the hand of physicians in the treatment of DME with its features such as reducing the number of injections due to its longer-lasting effect compared to anti-VEGF agents and being effective in different steps that cause DME formation. Accordingly, it is a good treatment alternative in patients with persistent macular edema and unresponsive to anti-VEGF agents [**6**]. A total of twenty-eight applications were made for dexamethasone implant in 2018. A similar study conducted in Turkey in 2013 reported that a total of 320 applications were made for the dexamethasone implant, which was not licensed and reimbursed at that time. Since dexamethasone implant obtained a license for the treatment of DME in 2016, the number of off-label use applications decreased significantly in 2018 [**19**].

The Diabetic Retinopathy Clinical Research Network (DRCR.net) recommends anti-VEGF drugs for DME in the first place [**20**]. These drugs have been shown to be effective against vision-threating complications. However, in some patients, repeated anti-VEGF applications may lead to a decrease in drug response due to tachyphylaxis and tolerance development [**21**]. To surmount this, switching to another anti-VEGF drug may be considered due to the different molecular structures and mechanisms of action of anti-VEGF drugs [**6**,**16**,**22**]. In the clinical practice of 2018 in Turkey, after the first switch between ranibizumab and aflibercept when it was necessary to switch back to the previous drug, an application for off-label drug use was mandatory. Therefore, in our study, the most common reason for off-label applications for ranibizumab/ aflibercept treatments was the request for a switchback.

The limitations of our study were the retrospective study design and the inability to provide data on the use of bevacizumab and triamcinolone because they did not require permission.

## Conclusion

In conclusion, the data we presented in our study provide information about the most preferred off-label treatment options and the previous and current drug regulations in the treatment of DRP in Turkey. Intravitreal drug treatments impose severe economic and time-consuming burden on healthcare systems. Although bevacizumab is not an approved drug in the treatment of DRP, it is frequently preferred because it is much more cost-effective than approved drugs. Therefore, in the current regulation in our country, initiating treatment with three doses of bevacizumab is mandatory. As far as we know, there is no study in the English literature regarding the off-label use of approved drugs such as aflibercept, ranibizumab and dexamethasone implant in the treatment of DRP. In this regard, the data presented in this study can serve as a model for drug regulatory authorities and healthcare professionals in other countries.

**Conflict of Interest statement**

The authors state no conflict of interest.

**Informed Consent and Human and Animal Rights statement**

Informed consent has been obtained from all individuals included in this study.

**Authorization for the use of human subjects**

Ethical approval: The research related to human use complies with all the relevant national regulations, institutional policies, is in accordance with the tenets of the Helsinki Declaration, and has been approved by the Ethical Committee of Ankara Diskapi Training and Research Hospital, Turkey.

**Acknowledgements**

None.

**Sources of Funding**

The authors declare that no funds, grants, or other support were received during the preparation of this manuscript.

**Disclosures**

The authors have no relevant financial or non-financial interests to disclose.
